# Robotic modified Appleby: Case report, technical aspects and video^☆^

**DOI:** 10.1016/j.ijscr.2022.107266

**Published:** 2022-06-01

**Authors:** Guilherme De Andrade Gagheggi Ravanini, Saskia Barreto De Almeida, Dyego Sá Benevenuto, Amanda Dal Castel Ferreira Da Silva, Fernanda Neres Ribeiro De Lima, Beatriz Escudeiro Nascimento

**Affiliations:** a Department of Surgery, Division of Surgical Oncology, Gaffrée e Guinle University Hospital, Federal University of Rio de Janeiro State (UNIRIO), Brazil; b Federal University of the State of Rio de Janeiro (UNIRIO), Brazil; c Department of General Surgery, Hospital Copa D'or, Rio de Janeiro, Brazil

**Keywords:** Pancreatectomy, Robotic surgery, Pancreatic neoplasms

## Abstract

**Introduction and importance:**

Pancreatic body and tail adenocarcinoma is a tumor of silent evolution, showing up as advanced disease at diagnosis in the majority cases.

**Case presentation:**

A 60-year-old man, presenting a solid expansive lesion (3.1 × 2.8 cm) in the body of the pancreas, by imaging exams. There was 50% involvement of the celiac trunk at the level of the it's bifurcation and suspected peripancreatic lymph nodes. Clinical staging was cT4N1M0. After neoadjuvant chemotherapy (NQT), there was a small reduction of the tumor to 3.0 × 2.6 cm, an important decreasing of the celiac trunk involvement (25%), and also a reduction in the levels of C19–9 from 650 U/mL to 358 U/mL. Further on, the patient underwent a Robotic Modified Appleby procedure, without intra and postoperative complications.

**Clinical discussion:**

Locally advanced pancreatic tumors should be referred to NQT, and those with good response might benefit to radical surgery. The modified Appleby technique - distal pancreatectomy with splenectomy and celiac trunk resection – is the procedure of choice when there is involvement of celiac trunk, but concern must be given to the liver vascularization. The use of robotic system on pancreatic surgery is emerging, but still, there is lower data regarding its safety in major pancreatic procedures.

**Conclusion:**

The use of neoadjuvant therapy is recommended to assess the in vivo response, improve oncological results and increase negative margins. Pre-operative imaging is mandatory for surgical planning. The robotic modified Appleby procedure is feasible and safe, with reduced of intraoperative blood loss and transfusion rate, corroborating the literature.

## Highlights

• 30% of pancreatic cancer presents as locally advanced disease at diagnosis with invasion to vascular structures.
• Arterial involvement is the biggest challenge for oncological resection, and involvement of the celiac axis or its branches can make curative resection impossible
• Robotic systems have been used in pancreatic surgery, but there is fewer data regarding feasibility and safety in major procedures.
• Preoperative radiology is crucial to assess the response to neoadjuvant therapy and allows for the analysis of vascular anatomy.

## 1 Introduction

Ductal adenocarcinoma is the most common histological subtype among the malignant neoplasms of the pancreas corresponding to more than 90% of primary pancreatic cancers [1]. Approximately 65% of these neoplasms occur in the head of the pancreas, 15% in the body/tail, and the remainder diffusely affect the organ [2]. In 30% of all cases, the disease is locally advanced at diagnosis with invasion to vascular structures and absence of distant metastases [2], [3], which tends to be more common with tumors located in the body/tail due to its insidious clinical nature. Arterial involvement is the biggest challenge for oncological resection, and involvement of the celiac axis or its branches can make curative resection impossible [3].

Locally advanced pancreatic tumors benefit from neoadjuvant therapy, and responding patients should undergo surgery. These patients receive NQT with FOLFIRINOX (folinic acid, fluorouracil, irinotecan and oxaliplatin), or Nab-Paclitaxel plus Gemcitabine regimen, with or without Stereotactic Body Radiation Therapy (SBRT). This strategy has been proven to be advantageous to in vivo assessment of chemosensitivity, selecting individuals who may, in fact, benefit from subsequent surgical resection, preventing unnecessary procedures in patients with aggressive biological tumors. NQT was shown to be beneficial in reducing tumor size and vascular involvement – tumor downstaging – and increasing the possibility of a complete oncological resection with negative margins (R0), reducing local recurrence [4].

The Appleby procedure was first described by Lyon Appleby, in 1953, for the treatment of locally advanced gastric cancer, in which celiac trunk resection was performed in block, with total gastrectomy and distal pancreatectomy. The modified Appleby procedure (MAP) has been used for the treatment of T4 body and tail pancreatic cancer involving the celiac trunk or its branches, in which the stomach is preserved, but distal pancreatectomy, splenectomy and resection of the celiac trunk are performed [4]. From that, MAP gained some focus in recent years because of its potential to achieve radical resection with negative margins [4]. Recently, robotic systems have been used in pancreatic surgery, but there is fewer data regarding feasibility and safety in major procedures.

## 2 Case presentation

**Fig. 1:**
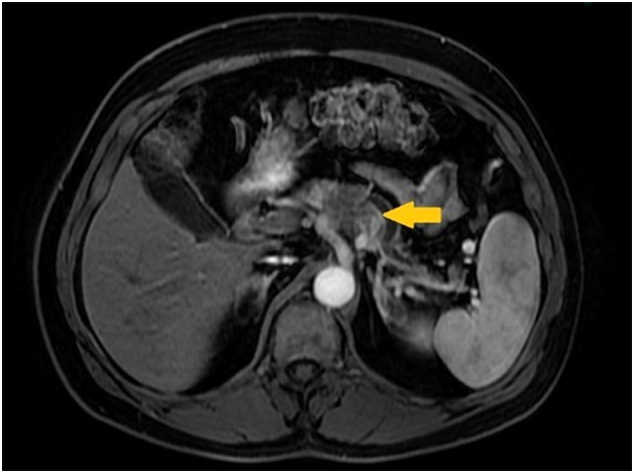
CT image before neoadjuvant chemotherapy. Nodular lesion (indicated by the yellow arrow) with hypovascular behavior measuring 3.1 × 2.8 cm in the body of the pancreas. (For interpretation of the references to colour in this figure legend, the reader is referred to the web version of this article.)

A 60-year-old man with controlled hypertension (Losartan 50 mg - daily) and no family history of genetic or oncological diseases presented abdominal discomfort for over 4 weeks. The physical examination results were unremarkable. A computed tomography (CT) revealed a 3,0 × 2,7 cm solid expansive lesion at the pancreatic body with contact to the celiac trunk. Magnetic resonance imaging (MRI) confirmed a nodular lesion with hypovascular behavior measuring 3,1 × 2,8 cm in the pancreas body (Fig. 1). The left distal portion of the celiac trunk, at the level of the bifurcation, had a 50% tumor contact, involving the splenic artery with a reduction in its caliber, without visualization of the splenic vein due to thrombosis. Peripancreatic lymph nodes were evident, the largest measuring 0.8 cm, suggesting involvement. CA19-9 marker was 650 U/mL. CT-guided percutaneous biopsy was performed and revealed a pancreatic ductal adenocarcinoma tumor with cT4N1M0 staging.

**Fig. 2:**
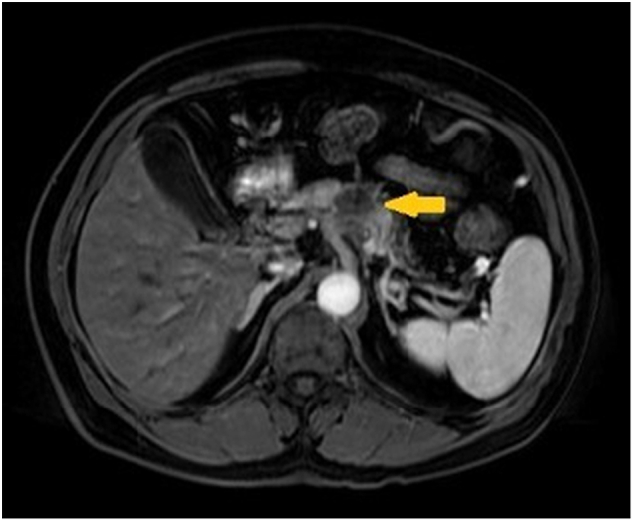
CT image after neoadjuvant chemotherapy. Small tumor reduction (indicated by the yellow arrow) measuring 3.0 × 2.6 cm. (For interpretation of the references to colour in this figure legend, the reader is referred to the web version of this article.)

Therefore, the patient received 6 cycles of neoadjuvant chemotherapy with FOLFIRINOX regimen allowing a small tumor reduction (3,1 × 2,8 to 3,0 × 2,6 cm), decreasing the contact with the left branch of the celiac trunk to 25% and also reducing C19-9 to 358 U/mL (Fig. 2). There was low toxicity during chemotherapy.

**Fig. 3:**
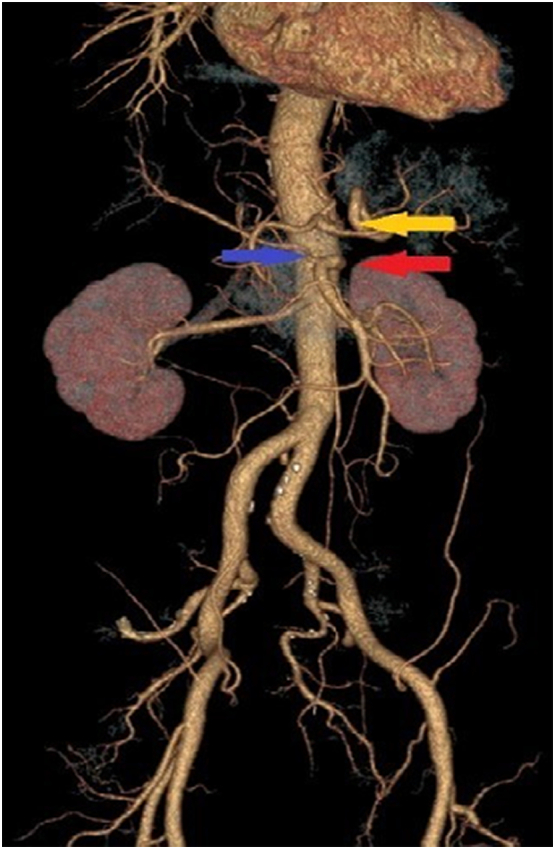
Three-dimensional reconstruction of computed angiotomography. The right hepatic artery – indicated by the blue arrow – is originated from the superior mesenteric artery – indicated by the red arrow. The yellow arrow indicates the emergence of the celiac trunk. (For interpretation of the references to colour in this figure legend, the reader is referred to the web version of this article.)

Abdominal angiotomography (Fig. 3) demonstrated an important anatomical variation that favored the procedure: the right hepatic artery originated from the superior mesenteric artery, the left hepatic artery originated from the left gastric artery and the common hepatic artery vascularized the IV segment (also called middle hepatic artery).

After restaging CTs and MRI he was referred for surgery – Robotic MAP with retroperitoneal lymphadenectomy and splenectomy. The procedure was performed by an experienced oncological surgeon. It lasted 6 h, with blood loss inferior to 200 ml, without blood transfusion and no perioperative complications. The Da Vinci Si System (Intuitive) was used.

On the 3rd postoperative day, there was an increase in hepatic enzymes (AST and ALT) above 700 U/L, with a progressive decrease in the two subsequent days. The abdominal drain was removed on the 5th postoperative day after amylase measurement, with hospital discharge on the same day.

After surgery, CA19-9 was 50 U/mL. The histopathological report showed a residual, regression 3, adenocarcinoma measuring 3.5 cm. Final staging ypT2ypN0 - T2: tumor with more than 2 cm restricted to the pancreas; N0: absence of metastases in regional lymph nodes. Six lymph nodes were isolated. Adjuvant chemotherapy was delivered on the following 6 months.

## 3 Surgical technique

Click here to access the surgery video.{https://drive.google.com/file/d/1E-amgIxevT3UO42Sw0Hukjllu-q1oQ4y/view?usp=sharing}

General and epidural anesthesia plus deep vascular access and arterial line were performed. Patient was placed in supine position with 30 degrees reverse-Trendelemburg, with both arms and legs open. Pneumoperitoneum was created with Veress needle at the left upper quadrant.

**Fig. 4:**
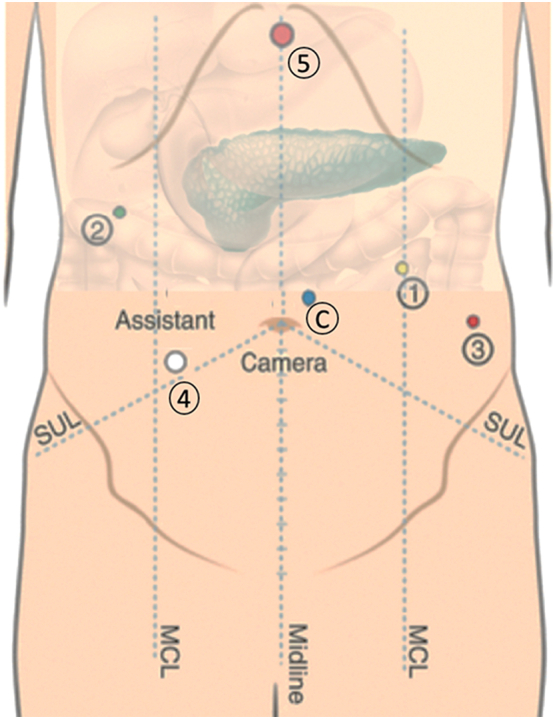
Trocar placement. C; Camera. 1; 8 mm first robotic arm. 2; 8 mm second robotic arm. 3; 8 mm third robotic arm. 4; 12 mm assistant port. 5; 5 mm liver retractor.

Port placement: The robotic camera was placed 2 cm above the umbilicus, slightly to the left. The first 8 mm arm was placed at the left middle clavicular line, 5 cm below the costal board. The second 8 mm arm was placed laterally to the right middle clavicular line, 2-4 cm below de costal board. The third 8 mm arm was placed laterally to the left middle clavicular line at the umbilicus height. A 12 mm auxiliary port was placed between arm 2 and camera port, 2 cm below de umbilicus for the assistant, and finally a 5 mm port was placed under the xiphoid process for the liver retractor (Fig. 4).

The Si da Vinci Robotic System was docked through the cephalic region and the assistant was positioned between the legs.

The procedure started with the release of the gastrocolic ligament and short gastric vessels for the positioning of the liver retractor below the stomach to elevate it and expose the retrocavity. Then, a retroperitoneal lymphadenectomy was performed through the dissection of the hepatic common artery and middle colic vessels (nodal stations 8 and 14 V). The first vascular approach was the ligation of the splenic artery, in order to reduce the portal flow and avoid excessive bleeding in case of portal vein injury. Then the left gastric vessels were ligated. The dissection of the portal vein tunnel was started under de pancreas, the common hepatic artery was ligated, so the upper board of the pancreas could be exposed and the portal vein dissection continued. The Warren maneuver (Portal vein dissection) was performed with careful and a cardiac tape was passed in the posterior portion of the pancreas to facilitate its traction and section. Pancreatic section was done with vascular mechanical stapling, and the remaining bleeding vessels were coagulated with robotic bipolar and ultrasound energy. After that, the gastric vein was recognized closer to its entrance to the portal vein, and was ligated again for *en bloc* resection. Dissection of the splenic vein was started and an absence of a cleavage plane was observed, suggesting tumor invasion. Therefore, dissection of the mesocolon was performed to ligate the inferior mesenteric vein. The dissection of the splenic vein and its ligation was performed with vascular stapling, just after the portosplenic confluence. The retroperitoneal lymphatic ducts were ligated to prevent lymphorrhea or chylous ascites. The ligation of the celiac trunk was done with vascular mechanical stapling in its aortic emergence. The rest of the pancreas and spleen were released from their attachments. A pancreatic monofilament suture was done at the stapling line, to prevent leakage. The pancreatic stump was protected with a hemostatic agent (reducing the chance of fistulae and intraperitoneal hemorrhage) and, finally, the cavity was drained. The specimen was removed by transumbilical counter-incision, with wound retractor.

## 4 Discussion

For years, systemic metastases, retroperitoneal invasion or noble vessels involvement, such as the common hepatic artery and celiac trunk, characterized pancreatic tumors as unresectable. However, the frequent involvement of vessels, such as the celiac trunk, has led in recent decades, to the improvement of the pancreatic surgical approach through the modified Appleby procedure. Thus, after rigorous selection of patients, some lesions could be resected with safe and improved results [6].

Despite being a longer procedure and associated with more blood loss and blood transfusion compared with conventional distal pancreatectomy (DP), when performed by the robotic approach the MAP resulted in reduced intraoperative blood loss and transfusion rate [3].

In spite of the longer surgical time compared to laparoscopic DP [7], the robotic platform allows more delicate and precise movements, in addition to an enlarged and 3D image. The postoperative period of robotic MAP is less painful, has a shorter hospital stay and a quick return to usual activities. In addition, better esthetic result and reduction in the infection rate in comparison to the conventional procedure [8]. On the other hand, it presents a 35% rate of postoperative complications [7], such as: pancreatic fistula (58.8%), ischemic gastropathy (28.8%) and delayed gastric emptying (20%). These complications were not found at the presented case, probably because there was a high attention to the pancreatic stump, and the anatomical vascular variation, which guaranteed a good hepatic blood supply.

Due to the radical nature of the technique and possible postoperative complications, some authors have proposed specific indications for its performance: no invasion of the pancreas head, superior mesenteric artery or proper hepatic artery; resectability of the root of the celiac trunk and common hepatic artery before the removal of the gastroduodenal artery; possibility of complete retroperitoneal release; and confirmation of an adequate liver perfusion after clamping the common hepatic artery, which was possible in the case reported because of the anatomical variation described [9]. Furthermore, the use of neoadjuvant therapy is recommended to assess the in vivo response, improve radicality and increase the possibility of R0 resection (reduction in tumor size and vascular involvement).

Although it is not a widely accepted technique, individuals undergoing MAP have a longer survival than those without surgery (14 months and 5 months, respectively) and morbidity and mortality similar to those undergoing conventional DP or body and tail pancreatectomy. In both surgical modalities, operative mortality rates vary between 0 and 5%, depending on the experience of the surgical team [10].

Preoperative radiology is crucial to assess the response to neoadjuvant therapy and allows for the analysis of vascular anatomy, invasion or involvement of other structures, ensuring eligibility for the procedure. It is also important to carefully assess the patient's vascularization, paying attention to possible anatomical variations, as in the case reported here: right hepatic artery originating from the superior mesenteric artery (prevalence of 10%) [11]; left hepatic artery originating from the left gastric (prevalence of 9.7%) [12]; association of both variations, (prevalence of 2.3%) [12]; and vascularization of segment IV by the common hepatic artery (prevalence of 1% for vascularization of this segment by the proper hepatic artery) [13]. These variations favor the performance of the procedure, since the hepatic blood supply is maintained even with the resection of the celiac trunk, with no need for any type of vascular reconstruction. In absence of the variations reported, it's important to verify the vascular patency during the procedure. In this case, a bulldog clamp should be applied at the common hepatic artery prior to its ligation for at least 10 to 15 min, and the liver arterial supply should be observed. If the liver, or the gallbladder shows any signs of ischemia, a vascular reconstruction of the common hepatic artery should be performed.

It is essential that there is a prior selection of patients by a multidisciplinary committee, evaluating the patient's general conditions, response to neoadjuvant therapy, possibility of curative resection (R0) and team experience [10], in order to minimize morbidity and mortality rates and ensure greater success surgical.

## 5 Conclusion

The robotic MAP proved to be a safe option in the treatment of body/tail pancreatic cancer, with similar morbidity and mortality when compared to conventional DP, with reduction in intraoperative blood loss and the rate of transfusion, relevant advantages in accordance with the literature.

This work has been reported in line with the SCARE 2020 criteria [5].

The following are the supplementary data related to this article.

Video S1 Robotic Appleby pub final. Video S1

Supplementary material Image 1

## Abbreviations

NQT: Neoadjuvant chemotherapy
CT: Computed Tomography
MRI: Magnetic Resonance Imaging
MAP: Modified Appleby Procedure
DP: Distal Pancreatectomy
3D: Three-dimensional

## Consent for publication

Written informed consent was obtained from the patient for publication of this case report and accompanying images. A copy of the written consent is available for review by the Editor-in-Chief of this journal on request.

## Availability of data and materials

The datasets used and/or analysed during the current study are available from the corresponding author on reasonable request.

## Provenance and peer review

Not commissioned, externally peer-reviewed.

## Ethical approval

Not applicable.

## Funding

There was no funding for this case report.

## Guarantor

Ravanini, Guilherme de Andrade G.

## Research registration number

Not applicable.

## CRediT authorship contribution statement

Guilherme A. G. Ravanini - data collection, critically revising article, reviewed final version of article, creation of figures.

Saskia Barreto de Almeida - study concept, manuscript writing, revising article, reviewed final version of article, study oversight.

Dyego Sá Benevenuto - data collection, critically revising article, reviewed final version of article.

Amanda Dal Castel Ferreira da Silva, Fernanda Neres Ribeiro de Lima e Beatriz Escudeiro Nascimento - study concept, literature review, revising article.

## Declaration of competing interest

None declared by the author.

## References

[1] Tempero M.A., Malafa M.P., Al-Hawary M., Asbun H., Bain A., Behrman S.W., Benson A.B., Binder E., Cardin D.B., Cha C., Chiorean E.G., Chung V., Czito B., Dillhoff M., Dotan E., Ferrone C.R., Hardacre J., Hawkins W.G., Herman J., Ko A.H., Komanduri S., Koong A., LoConte N., Lowy A.M., Moravek C., Nakakura E.K., O’Reilly E.M., Obando J., Reddy S., Scaife C., Thayer S., Weekes C.D., Wolff R.A., Wolpin B.M., Burns J., Darlow S. (2017). Pancreatic adenocarcinoma, version 2.2017, NCCN clinical practice guidelines in oncology. J. Natl. Compr. Cancer Netw..

[2] Artinyan A., Soriano P.A., Prendergast C., Low T., Ellenhorn J.D.I., Kim J. (2008). The anatomic location of pancreatic cancer is a prognostic factor for survival. HPB.

[3] Cannella R., Borhani A.A., Zureikat A.H., Tublin M.E. (2019). Appleby procedure (Distal pancreatectomy with celiac artery Resection) for locally advanced pancreatic carcinoma: indications, outcomes, and imaging. Am. J. Roentgenol..

[4] Beane J.D., Zureikat A.H. (2018). Robotic distal pancreatectomy with celiac axis resection for locally advanced pancreatic cancer. Ann. Pancreat. Cancer.

[5] Agha R.A., Franchi T., Sohrabi C., Mathew G., Kerwan A., Thoma A., Beamish A.J., Noureldin A., Rao A., Vasudevan B., Challacombe B., Perakath B., Kirshtein B., Ekser B., Pramesh C.S., Laskin D.M., Machado-Aranda D., Miguel D., Pagano D., Millham F.H., Roy G., Kadioglu H., Nixon I.J., Mukherjee I., McCaul J.A., Chi-Yong Ngu J., Albrecht J., Rivas J.G., Raveendran K., Derbyshire L., Ather M.H., Thorat M.A., Valmasoni M., Bashashati M., Chalkoo M., Teo N.Z., Raison N., Muensterer O.J., Bradley P.J., Goel P., Pai P.S., Afifi R.Y., Rosin R.D., Coppola R., Klappenbach R., Wynn R., de Wilde R.L., Surani S., Giordano S., Massarut S., Raja S.G., Basu S., Enam S.A., Manning T.G., Cross T., Karanth V.K.L., Kasivisvanathan V., Mei Z., The S.C.A.R.E. (2020). Guideline: updating consensus surgical CAse REport (SCARE) guidelines. Int. J. Surg..

[6] Latona J.A., Lamb K.M., Pucci M.J., Maley W.R., Yeo C.J. (2016). Modified Appleby procedure with arterial reconstruction for locally advanced pancreatic adenocarcinoma: a literature review and report of three unusual cases. J. Gastrointest. Surg..

[7] Ocuin L.M., Miller-Ocuin J.L., Novak S.M., Bartlett D.L., Marsh J.W., Tsung A., Lee K.K., Hogg M.E., Zeh H.J., Zureikat A.H. (2016). Robotic and open distal pancreatectomy with celiac axis resection for locally advanced pancreatic body tumors: a single institutional assessment of perioperative outcomes and survival. HPB.

[8] Brazilian College of Surgeons [Internet] (2004). Surgery self-assessment program: minimally invasive surgery, diagraphic. https://cbc.org.br/wp-content/uploads/2013/05/Ano3-III.Cirurgia-minimamente-invasiva.pdf.

[9] Deal S., Nathan D., Rocha F.G. (2018). Modified Appleby procedure for locally advanced pancreatic cancer. Am. J. Surg..

[10] Sebastián J.López, del Castillo J.M.Gamez, Fernández Á.Castro, Corner E.Muñoz, Espinoza C.León, Ortí L.Sabater (2016). Body-caudal pancreatectomy with celiac trunk resection for pancreatic body adenocarcinoma: modified Appleby intervention. Rev. Méd. Uruguay.

[11] Sebben G.A., Rocha S.L., Sebben M.A., Parussolo Filho P.R., Gonçalves B.H.H. (2013). Variations of hepatic artery: anatomical study on cadavers. Rev. Col. Bras. Cir..

[12] Hiatt J.R., Gabbay J., Busuttil R.W. (1994). Surgical anatomy of the hepatic arteries in 1000 cases. Ann. Surg..

[13] Zaki S.M., Abdelmaksoud A.H.K., Khaled B.E.A., Abdel Kader I.A. (2020). Anatomical variations of hepatic artery using the multidetector computed tomography angiography. Folia Morphol. (Warsz).

